# Maternal Malnutrition Affects Hepatic Metabolism through Decreased Hepatic Taurine Levels and Changes in HNF4A Methylation

**DOI:** 10.3390/ijms21239060

**Published:** 2020-11-28

**Authors:** Ji Eun Du, Young Ah You, Eun Jin Kwon, Soo Min Kim, Jeongae Lee, Ki Hwan Han, Young Ju Kim

**Affiliations:** 1 Department of Obstetrics and Gynecology and Ewha Medical Research Institute, College of Medicine, Ewha Womans University, Seoul 07985, Korea; jedoo99@hanmail.net (J.E.D.); yerang02@naver.com (Y.A.Y.); friendkej1004@hanmail.net (E.J.K.); zeus_0218@naver.com (S.M.K.); 2 Molecular Recognition Research Center, Korea Institute of Science and Technology, Seoul 02792, Korea; frans@kist.re.kr; 3 Department of Anatomy, College of Medicine, Ewha Womans University, Seoul 07985, Korea; khhan@ewha.ac.kr

**Keywords:** fetal programming, food restriction, high fat diet, taurine, HNF4A methylation

## Abstract

Fetal programming implies that the maternal diet during pregnancy affects the long-term health of offspring. Although maternal diet influences metabolic disorders and non-alcoholic fatty liver disease in offspring, the hepatic mechanisms related to metabolites are still unknown. Here, we investigated the maternal diet-related alterations in metabolites and the biological pathway in male offspring at three months of age. Pregnant rats were exposed to 50% food restriction during the prenatal period or a 45% high-fat diet during the prenatal and postnatal periods. The male offspring exposed to food restriction and high-fat diets had lower birth weights than controls, but had a catch-up growth spurt at three months of age. Hepatic taurine levels decreased in both groups compared to controls. The decreased hepatic taurine levels in offspring affected excessive lipid accumulation through changes in hepatocyte nuclear factor 4 A methylation. Moreover, the alteration of gluconeogenesis in offspring exposed to food restriction was observed to a similar extent as that of offspring exposed to a high fat diet. These results indicate that maternal diet affects the dysregulation in hepatic metabolism through changes in taurine levels and HNF4A methylation, and predisposes the offspring to Type 2 diabetes and non-alcoholic fatty liver disease in later life.

## 1. Introduction

The maternal diet during pregnancy influences fetal development and the long-term health of offspring [1]. Maternal under-nutrition or over-nutrition during pregnancy causes reduced placental-fetal blood flow and intrauterine growth restriction (IUGR) [2,3]. A low birth weight due to IUGR is associated with an increased risk of obesity and metabolic syndrome [4,5]. Thus, considerable attention is being focused on the link between the intrauterine environment and adult diseases such as diabetes, obesity, hypertension, metabolic disease, and nonalcoholic fatty liver disease (NAFLD) [6,7]. However, the molecular mechanisms responsible for the association between maternal food restriction or high fat diet and fetal development/long-term health are still unknown.

The liver is a central organ in cell growth and metabolic processes including lipogenesis and gluconeogenesis. Several studies have suggested that suboptimal maternal nutrition during pregnancy leads to increased hepatic lipid accumulation in the offspring [8,9,10]. Moreover, in animal models, maternal macronutrient restriction has been shown to affect liver development and induce susceptibility to NAFLD in later life [11]. The pathogenesis of NAFLD involves dysfunction of the hepatic lipid metabolism process, oxidative stress, changes in the microbiome, and DNA damage. Poor maternal nutrition may cause fetal growth retardation and permanent changes in key organs associated with glucose homeostasis [12]. Epidemiological studies show that a suboptimal environment during fetal development affects carbohydrate metabolism rates and susceptibility to later development of metabolic disorders including insulin resistance and hypertension [13,14]. The dysregulation of these processes affects fetal liver development and increases metabolic disorders in later life [15].

Metabolomics is used to evaluate relative changes in the levels of metabolites, and could lead to the identification of new biomarkers including lipids, sugars, and amino acids to diagnose and predict disease [16,17]. A recent study suggested that some metabolites are altered in clinical and animal samples of NAFLD, IUGR, and obesity [18,19,20]. Thus, metabolomics plays a critical role in the mechanism behind metabolic diseases resulting from fetal programming such as obesity, NAFLD, and the metabolic syndrome.

In this study, we aimed to identify the differences in plasma metabolomics profiles of rat male offspring according to maternal nutrition. We focus on determining the biological pathway underlying the association between maternal diet and hepatic lipogenesis and gluconeogenesis.

## 2. Results

### 2.1. Characteristics of 3-Month-Old Male Offspring

The body weight of male offspring was measured for all three groups during the study period (ad libitum (AdLib)/AdLib, food restriction (FR)/AdLib, and high-fat (HF)/HF) at the time of birth and at the age of 3 months. The weight gain of male offspring at 3 months of age was higher in the FR/AdLib and HF/HF groups compared to controls, while body weight at birth was significantly lower than the control group (*p* < 0.05). Food intake in the FR/AdLib and HF/HF groups was lower than in their control, but there were no significant differences among three groups. Whereas food efficiency ratio (FER) of FR/AdLib and HF/HF group was significantly increased compared to control (*p* < 0.05, Figure S1). The liver and epididymal fat-pad weight was significantly higher in the HF/HF group compared to controls; however, there was no significant difference between the control group and FR/AdLib group (*p* < 0.05, Table 1).

At 3 months of age, the fasting glucose levels and the homeostasis model assessment for insulin resistance (HOMA-IR) values significantly increased in the FR/AdLib and HF/HF groups compared to controls (*p* < 0.05). In addition, the levels of insulin and total cholesterol (TC) significantly increased in the HF/HF group compared to controls (*p* < 0.05, Table 2).

### 2.2. Metabolomic Analysis in Plasma in 3-Month-Old Male Offspring

**Figure 1:**
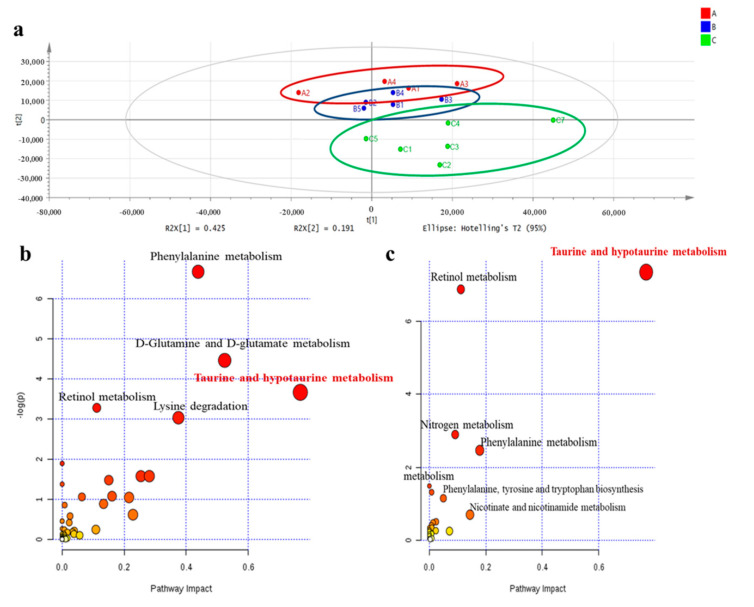
Comparison of plasma metabolic profiles according to maternal diet in male offspring at 3 months of age. (**a**) PLS-DA scatterplot of plasma from offspring according to maternal diet to identify metabolites that contributed to differences among the three groups, based on mass spectrometry using the loading plot from these graphs; Red color, control; Blue color, FR/AdLib; Green color, HF/HF. (**b**) Metabolites were altered significantly in the FR/AdLib group compared to controls. (**c**) The metabolites were altered significantly in the HF/HF group compared to controls. The most significant metabolic pathways were represented as circles in different sizes and colors; larger circles or circle in darker colors indicated higher-impact metabolic pathways (yellow < orange < red); AdLib, ad libitum; FR, food restriction; HF, high fat; PLS-DA, partial least squared discriminate analysis.

**Figure 2:**
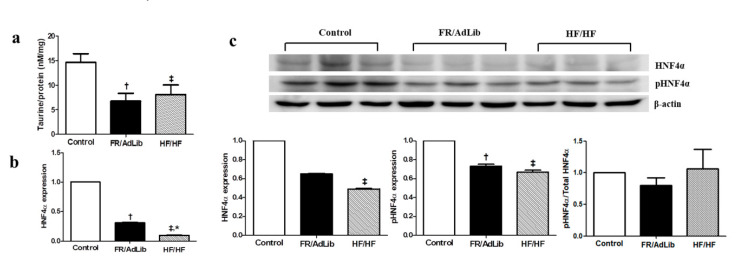
The levels of hepatic taurine and relative mRNA and protein expression of HNF4A in the liver of male offspring at 3 months of age (*n* = 9, control; *n* = 9, FR/AdLib; *n* = 9 HF/HF). (**a**) The levels of hepatic taurine were determined by ELISA. (**b**) The levels of mRNA encoding HNF4A were determined by real-time PCR. (**c**) The protein expression levels of HNF4A were measured using Western blotting. Values are presented as the mean ± SEM. *p*-values were calculated using one-way analysis of variance (ANOVA) followed by Tukey’s post-hoc test. † Control vs. FR/AdLib, ‡ Control vs. HF/HF, * FR/AdLib vs. HF/HF (*p* < 0.05). AdLib, ad libitum; FR, food restriction; HF, high fat; HNF4A, hepatocyte nuclear factor 4 A.

We conducted non-targeted metabolomics to quantify the metabolite difference between controls and our experimental groups in the plasma of male offspring at 3 months of age. The partial least squared discriminate analysis (PLS-DA) score plots showed that the metabolomics pattern for FR/AdLib and HF/HF was clearly distinguished from that for control. The levels of several metabolites were significantly altered in the FR/AdLib group compared to controls, especially with regard to phenylalanine metabolism rates, D-glutamine and D-glutamate metabolism rates, and the retinol metabolism pathway (*p* < 0.05). Compared to controls, the HF/HF group exhibited significantly altered levels of metabolites via retinol metabolism, nitrogen metabolism, and phenylalanine metabolism (*p* < 0.05). In particular, taurine and hypotaurine metabolism rates were significantly altered in both the FR/AdLib and HF/HF groups (*p* < 0.05, Figure 1). We observed that levels of hepatic taurine significantly decreased in the FR/AdLib and HF/HF groups compared to the control group (*p* < 0.05, Figure 2a).

### 2.3. Hepatic HNF4A Methylation and Expression in 3-Month-Old Male Offspring

We determined the expression levels of several hepatic genes related to the taurine pathway which regulate hepatic lipid metabolism rates. Among these hepatic genes, only the mRNA levels of hepatocyte nuclear factor 4 A (HNF4A) was significantly altered compared with that in the control (*p* < 0.05, Figure S2). The mRNA expression level of HNF4A was significantly decreased in the FR/AdLib and HF/HF compared with that in the control group of 3-month-old male offspring (*p* < 0.05). The protein expression of phospho-HNF4A was significantly decreased in FR/AdLib and HF/HF compared with that in the control, while phosphor-HNF4A/total-HNF4A ratio had no significant difference among the three groups (*p* < 0.05, Figure 2). In addition, according to the maternal diet during pregnancy, the extent of HNF4A methylation’s effect on the P1 promoter was significantly high in both the FR/AdLib and HF/HF groups compared to the control group (*p* < 0.05, Table 3).

### 2.4. Hepatic Histology and Hepatic Triglyceride Analysis

**Figure 3:**
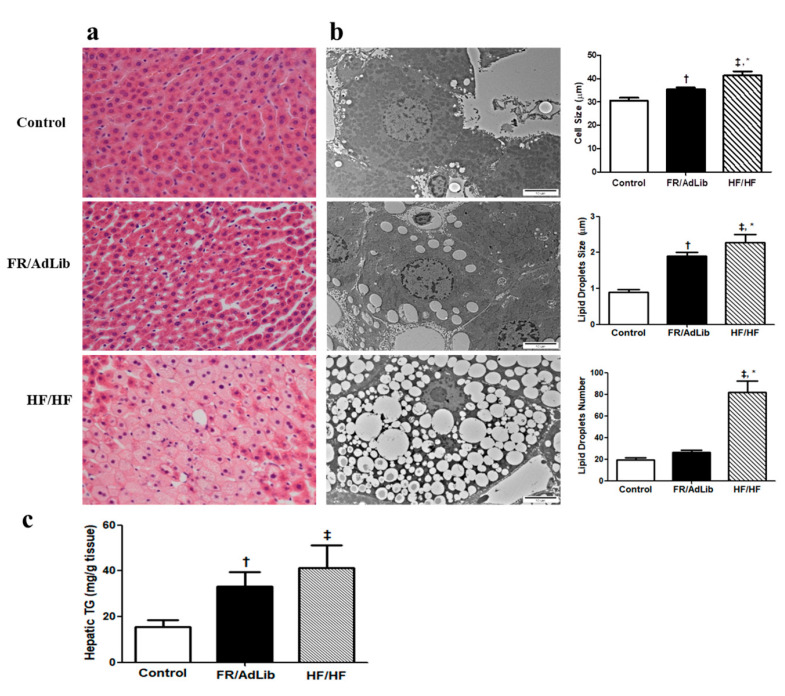
(**a**) Histopathological analysis of liver. Liver tissues from 3-month-old male offspring stained with hematoxylin and eosin (H and E). Original magnification (×400) (**b**) Analysis of lipid droplets and cell size in the liver using electron microscopy (×10,000). (**c**) The levels of hepatic triglyceride in the liver of male offspring. Values are presented as the mean ± SEM. *p*-values were calculated using one-way analysis of variance (ANOVA) followed by Tukey’s post-hoc test. † Control vs. FR/AdLib, ‡ Control vs. HF/HF, * FR/AdLib vs. HF/HF (*p* < 0.05). AdLib, ad libitum; FR, food restriction; HF, high fat; TG, triglyceride.

Hematoxylin and eosin (H and E) staining of the liver showed wider spaces between the hepatic cells and greater lipid accumulation in the FR/AdLib and HF/HF groups compared to controls (Figure 3a). To determine the size and number of the lipid droplets (LDs), we measured the diameter and number of LDs using electron microscopy. The LDs number was significantly increased in HF/HF (*p* < 0.05), while the cell size and LDs size significantly increased in both the FR/AdLib and HF/HF groups compared to controls (*p* < 0.05, Figure 3b).

We analyzed hepatic triglyceride (TG) using ELISA in the male offspring aged 3 months. The levels of hepatic TG significantly increased in the FR/AdLib and HF/HF groups compared to controls (*p* < 0.05, Figure 3c).

### 2.5. Maternal Diet Altered Hepatic Lipogenesis and Gluconeogenesis

**Figure 4:**
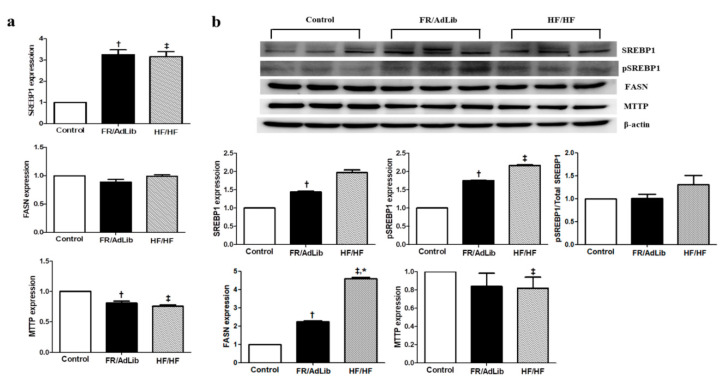
Relative mRNA and protein expression of SREBP1, FASN, and MTTP in the liver of male offspring at 3 months of age. (**a**) The levels of mRNA encoding SREBP1, FASN, and MTTP were determined by real-time PCR for the three groups (*n* = 9, control; *n* = 9, FR/AdLib; *n* = 9, HF/HF). (**b**) The protein expression levels of SREBP1, pSREBP1, FASN, and MTTP were measured using Western blotting for the three groups (*n* = 9, control; *n* = 9, FR/AdLib; *n* = 9, HF/HF). Values are presented as the mean ± SEM. *p*-values were calculated using one-way analysis of variance (ANOVA) followed by Tukey’s post-hoc test. † Control vs. FR/AdLib, ‡ Control vs. HF/HF, * FR/AdLib vs. HF/HF (*p* < 0.05). AdLib, ad libitum; FR, food restriction; HF, high fat; SREBP1, sterol regulatory element-binding protein 1; FASN, fatty acid synthase; MTTP, microsomal triglyceride transfer protein.

We determined the effect of maternal diet on hepatic lipogenesis by analyzing the expression of various genes involved in this process. The mRNA and protein expression of sterol regulatory element-binding protein 1 (SREBP1) significantly increased in the FR/AdLib group and the mRNA expression increased in the HF/HF group, compared to controls (*p* < 0.05). Moreover, the protein expression of phospho-SREBP1 significantly increased in the FR/AdLib and HF/HF groups compared to controls, while the phosphor-SREBB1/total-SREBP1 ratio had no significant difference among the three groups (*p* < 0.05). The protein expression of fatty acid synthase (FASN) significantly increased in the FR/AdLib and HF/HF groups compared to controls (*p* < 0.05). In the FR/AdLib group, the mRNA expression of microsomal triglyceride transfer protein (MTTP) significantly decreased, whereas both mRNA and protein expressions significantly decreased in the HF/HF group (*p* < 0.05, Figure 4).

**Figure 5:**
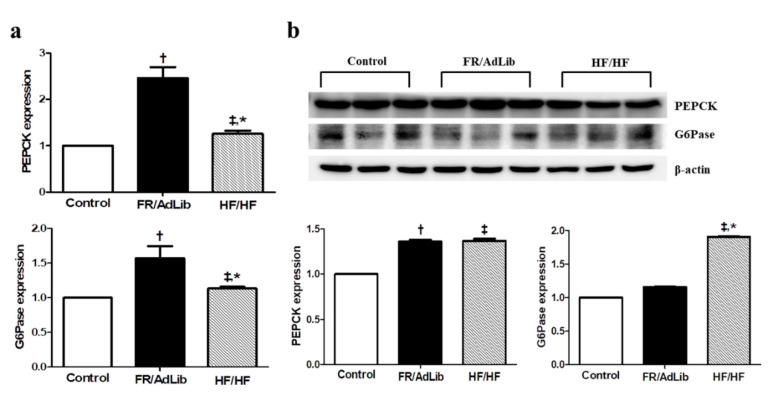
Relative mRNA and protein expression of PEPCK and G6Pase in the liver of male offspring at 3 months of age. (**a**) The levels of mRNA encoding PEPfCK and G6Pase were determined by real-time PCR in the three groups (*n* = 9, control, *n* = 9; FR/AdLib; *n* = 9, HF/HF). (**b**) The protein expression levels of PEPCK and G6Pase were measured using Western blotting in the three groups (*n* = 9, control; *n* = 9, FR/AdLib; *n* = 9, HF/HF). Values are presented as the mean ± SEM. *p*-values were calculated using one-way analysis of variance (ANOVA) followed by Tukey’s post-hoc test. † Control vs. FR/AdLib, ‡ Control vs. HF/HF, * FR/AdLib vs. HF/HF (*p* < 0.05). AdLib, ad libitum; FR, food restriction; HF, high fat; PEPCK, phosphoenolpyruvate carboxykinase; G6Pase, glucose-6-phosphatase.

We also determined the effect of maternal diet on hepatic gluconeogenesis. The mRNA and protein expression of phosphoenolpyruvate carboxykinase (PEPCK) was significantly increased in both FR/AdLib and HF/HF compared to that in the control (*p* < 0.05). In addition, the mRNA expression of glucose-6-phosphatase (G6Pase) was significantly increased in FR/AdLib and HF/HF compared to that in the control, while the protein expression was significantly increased only in HF/HF (*p* < 0.05, Figure 5).

## 3. Discussion

The maternal diet during the perinatal period influences fetal adaptive growth and liver metabolism. In the present study, male offspring born to mothers exposed to food restriction and a high fat diet showed a catch-up growth spurt, as well as higher plasma glucose levels compared to controls at three months of age. We found that maternal food restriction affects hepatic taurine and TG levels, and the accumulation of lipid droplets in offspring to a similar extent to that of offspring exposed to a high fat diet. Moreover, we observed that maternal food restriction and a high fat diet affects the dysregulation of hepatic lipogenesis through changes in HNF4A methylation and gluconeogenesis. This study indicates that manipulation of the maternal diet during pregnancy leads to dysregulation of hepatic metabolism and a predisposition to NAFLD and type 2 diabetes in later life.

In a Dutch Hunger Winter Study, the impact of prenatal famine exposure was demonstrated by an increased risk in obesity and metabolic disease among children of women exposed to famine during gestation. Therefore, epidemiological and animal studies have demonstrated the association between maternal diet and IUGR [3,6,7,21]. In addition, animal models of IUGR, particularly those who showed rapid catch-up growth, demonstrate an increased risk of obesity and metabolic syndrome induced by maternal malnutrition [4,22,23]. In our previous studies, we demonstrated that the offspring born to mothers exposed to food restriction during pregnancy showed catch-up growth and obesity at 3 weeks [24,25]. Moreover, the Hertfordshire Cohort Study showed that the birth weight and glucose intolerance had a negative association [26]. Therefore, we explored the effect of maternal food restriction on offspring at 3 months and compared it with a diet-induced obese model. In our study, the male offspring of mothers fed a food restriction or high fat diet showed catch-up growth. The fasting glucose levels and HOMA-IR values increased in both FR/AdLib and HF/HF compared to that in the control. Thus, this study indicates that maternal food restriction or high fat diet during pregnancy could result in susceptibility to later development of type 2 diabetes and metabolic disease.

Human and animal studies suggest that the alteration in the levels of certain metabolites are associated with obesity and metabolic syndrome; these observations could lead to the identification of new biomarkers through metabolomics to predict disease [16,17,18]. Thus, we explored this to compare metabolites according to maternal diet in the plasma. Our results showed that taurine and hypotaurine metabolism was significantly altered in both FR/AdLib and HF/HF compared to that in the control. Taurine plays an important role in maintaining normal lipid metabolism and has biological functions such as antioxidant and bile acid conjugation. Some studies suggest that taurine supplementation could prevent obesity and insulin resistance. Moreover, the hepatic taurine levels could influence hepatic steroid metabolism and other lipid metabolism [27,28,29]. Thus, we observed the hepatic taurine levels to determine the effect of hepatic taurine on lipid metabolism. In our result, the hepatic taurine levels were decreased in both FR/AdLib and HF/HF compared to that in the control. In the liver, taurine conjugates with bile acids, and it is secreted into the bile to regulate lipid metabolism. Several hepatic genes, such as cholesterol 7α-hydroxylase (CYP7A1), farnesoid X receptor (FXR), and HNF4A, are involved in bile acid biosynthesis and conjugation. Alteration of these pathways induces fatty liver disease, diabetes, and obesity [30,31].

In our results, among the hepatic genes associated with the taurine pathway, only the mRNA levels of HNF4A and protein levels of phospho-HNF4A significantly decreased in both FR/AdLib and HF/HF groups compared to controls. Because the ratio of phopho-HNF4A/total-HNF4A had no significant difference among the three groups, this result suggests that food restriction affects the level of the hepatic HNF4A protein in offspring. HNF4A is known to be associated with hepatic growth and lipid and glucose metabolism [32,33]. Several studies showed that loss of HNF4A function is associated with liver dysfunction [34]. In addition, the maternal diet during pregnancy induces DNA methylation changes, which negatively influences gene expression [35,36]. Consistently, our results reveal that the methylation status was altered across all CpG sites of hepatic HNF4A in both FR/AdLib and HF/HF compared to that in the control. Thus, this study indicates that manipulation of the maternal diet could lead to an alteration in the methylation level and expression of HNF4A through changes in the taurine pathway.

To determine the effects of HNF4A on lipid metabolism rates with respect to differences in the maternal diet, we observed LDs using an electron microscope. Our results showed that maternal food restriction increased the hepatic cell size and accumulation of LDs in the liver; however, the liver weight and plasma TG levels in these offspring were not different from that of the control. In addition, hepatic TG levels significantly increased, similar to that observed in the offspring of obese mothers. Over-accumulation of TG and LDs leads to obesity and NAFLD [37]. This study suggests the possibility that an insufficient maternal diet causes lipid accumulation and that it could lead to NAFLD in later life, similar to the effect of maternal high fat diet.

Increasing evidence suggests that NAFLD may result from the dysregulation of hepatic TG synthesis and secretion [8,9,10]. Hepatic transcription factors influence complex metabolic pathways and play a critical role in NAFLD [38]. SREBP1 regulates de novo lipogenesis and stimulates FASN, which is involved in de novo fatty acid synthesis [39,40]. TGs are assembled and secreted as very low-density lipoprotein (VLDL). MTTPs are essential for the assembly and secretion of VLDL [41,42]. HNF4A regulates the lipid transport protein and apolipoproteins [43]. Our results showed that the expression levels of SREBP1 and FASN, associated with TG synthesis, were increased, while the levels of MTTP, associated with secretion, were decreased compared to those in the control. Therefore, the current data indicates that the increased lipid synthesis caused by elevated SREBP1 and FASN and decreased lipid export caused by dysfunction of HNF4A and reduction in MTTP gene expression could lead to an over-accumulation of lipids and susceptibility to NAFLDs in later life.

Several studies suggest that gluconeogenesis increase in IUGR because glucose plays an important role as a major substrate in fetal metabolism and growth [44,45]. Moreover, according to Laplant et al., lipogenesis and gluconeogenesis were increased in the insulin-resistance model [46]. Consistently, our results show that the plasma fasting glucose level and HOMA-IR values in the offspring of mothers exposed to food restriction is increased to a similar extent to that in the offspring of mothers exposed to a high fat diet. In addition, the expression levels of PEPCK and G6Pase, which are involved in the key step of gluconeogenesis, were elevated compared to that in the control. Thus, these results indicate that male offspring exposed to food restriction during the prenatal period show elevated gluconeogenesis with increased lipogenesis and hepatic TG level and that they may exhibit insulin-resistance in later life.

This study had some limitations. We did not evaluate the effect of maternal diet on female offspring and transgenerational effects. Thus, further studies are needed to determine gender difference and long-term effects. Despite these limitations, this study has the strength of comparing lipid and glucose metabolism in the liver among three groups exposed to different maternal diets.

Our study suggests that although maternal food restriction during pregnancy did not alter the liver weight and plasma lipid profiles of offspring compared to control, it could lead to catch-up growth like offspring exposed to maternal high fat diet, and dysfunction in lipid and glucose metabolism in the liver. Moreover, the alteration of hepatic lipogenesis and gluconeogenesis during the postnatal period predisposes the offspring to NAFLD and type 2 diabetes in later life.

## 4. Materials and Methods

### 4.1. Animals and Study Design

Eight-week-old male and female Sprague-Dawley (SD) rats were purchased from Orient Bio (Orient Bio Inc, Seongnam, Kyunggi-do, Korea) and were exposed to 12/12 h cycle at a constant temperature (22 ± 2 °C) and humidity (55 ± 10%) during study period. The rats were mated after a one-week acclimation period with non-purified standard laboratory chow (Purina, Pyeongtaek, Korea). Pregnant rats were randomly divided into three groups on Day 10 of gestation: (1) AdLib/AdLib for control (fed ad libitum during pregnancy and lactation); (2) FR/AdLib (fed a 50% food-restriction diet during pregnancy and ad libitum during lactation); and (3) HF/HF (fed a 45% high-fat diet during pregnancy and lactation). Pregnant rats in each group gave birth to about 13 offspring each. After the weaning period, 9 offspring were selected in each group according to their diet, excluding those that were too small or overweight. The diet composition of standard laboratory chow, which was served as Adlib, FR, and 45% high-fat diet (Research Diets, New York, NJ, USA) are described in Figure S3. After delivery, the pups remained with their dams until lactation. During the study period, the body weight was measured at birth and weekly for up to 3 months (Figure S4). At the age of 3 months, the offspring were subjected to fasting overnight and sacrificed by exsanguination under Zoletil anesthesia (Virbac, Taguig, Philippines). Blood samples were collected immediately, and the weights of the liver and epididymal fat pad were measured. The samples were stored at −80 °C. Male offspring from the study groups AdLib/AdLib (*n* = 9), FR/AdLib (*n* = 9), and HF/HF (*n* = 9) were chosen randomly. This study was approved by the Animal Research Committee of the School of Medicine at Ewha Womans University (ESM16-0363, 16 January 2017) and was performed in accordance with the International Guidelines of Laboratory Care for Animals.

### 4.2. Plasma and Hepatic Biochemical Analysis

Blood samples were collected via cardiac puncture into heparinized tubes (BD Biosciences, San Jose, CA, USA) for biochemical profiling. The plasma was separated from the blood samples by centrifugation at 3000 rpm for 10 min and stored at −80 °C until biochemical analysis. The plasma levels of glucose, total cholesterol (TC), high-density lipoprotein (HDL)-cholesterol, low-density lipoprotein (LDL)-cholesterol and triglyceride (TG) levels were analyzed using an enzymatic colorimetric method with Cobas 8000 (Roche, Mannheim, Germany). Insulin was measured using an insulin ELISA kit (ALPCO, Salem, MA, USA) according to the manufacturer’s instructions, with the aid of a VersaMax ELISA Microplate Reader (Molecular Devices, San Jose, CA, USA). Hepatic TG and taurine concentrations were measured using a triglyceride assay kit (Abcam, Cambridge, UK) and taurine assay kit (Cell Biolabs, San Jose, CA, USA). The homeostasis model assessment (HOMA) for insulin resistance (IR) was calculated using the following formula (fasting plasma glucose [mmol/L] × fasting insulin [mU/L])/22.5 [47].

### 4.3. Plasma Metabolomics Analysis

The liquid chromatography mass spectrophotometry (LC-MS) analysis was performed using Ultimate 3000 ultra-high-performance liquid chromatography system coupled with a linear ion trap-Orbitrap mass spectrometer (UPLC-LTQ-Orbitrap MS) from Thermo Fisher Scientific (San Jose, CA, USA). Plasma samples of male offspring aged 3 months were deproteinized and homogenized, and extracted amples were applied to reversed-phase separation, performed on an Acquity™ UPLC BEH C18 column (2.1 mm × 100 mm, 1.7 μm, Waters, Milford, MA, USA) UPLC analytical column. The mobile phase solvents were 95% water, 5% ACN and 0.1% formic acid (mobile phase A) and 95% ACN, 5% water and 0.1% formic acid (mobile phase B). The elution gradient was as follows: 100% mobile phase A from 0 to 3 min; linear increase to 50% mobile phase B from 3 to 10 min; linear increase in mobile phase B from 50% to 90% from 10 to 12 min; maintainance of mobile phase B for 90% 12 to 12.5min; re-equilibration with 100% mobile phase A from 12.5 to 14 min. The column was maintained at 40 °C; total run time was 14 min. The samples were kept at 4 °C in an auto sampler during the analysis. Multivariate analysis (MVA) was performed using SIMCA-*P* software v14.0+ from Umetrics (Umeå, Sweden) for the principal component analysis (PCA), projection to latent structures discriminant analysis (PLS-DA) and orthogonal projection to latent structures-discriminant analysis (OPLS-DA). Pathway impact analysis was performed by Metaboanalyst 3.0 (Montréal, QC, Canada).

### 4.4. Hepatic Histological Analysis

For paraffin histology, the collected liver tissues were fixed in 10% formalin for 24 h. After fixation, the tissues were embedded into paraffin, sectioned into 4 μm thick slices, and placed on glass slides. The sections were deparaffinized and stained with hematoxylin and eosin (H and E). The sections were examined using a light microscope at 400× magnification. For electron microscopy, the liver tissues were perfused with 2% glutaraldehyde and 2% paraformaldehyde in 0.1 M phosphate buffer. The prepared blocks of liver were post-fixed in 2% osmium tetroxide, dehydrated, and embedded in epoxy-resin. The area of interest in the section, approximately 1 µm thick, was stained with toluidine blue, and ultra-thin sections of approximately 60–70 nm thickness, were cut using an ultramicrotome (Leica, Vienna, Austria) with a diamond knife. The thin sections were stained with 2% aqueous uranyl acetate, followed by 6.18% lead citrate. The sections were observed using an H-7650 transmission electron microscope (Hitachi, Tokyo, Japan) at an accelerating voltage of 80 kV and 10,000× magnification. The hepatic cell size and lipid droplet (LD) size and number were measured in 20 fields for each liver sample using SIS (Soft Imaging System GMBH, Munster, Germany).

### 4.5. DNA Methylation Analysis by Bisulfite Amplicon Sequencing

Genomic DNA was extracted from the liver of the male offspring at 3 months of age using a DNeasy Blood and Tissue Kit (Qiagen, Hilden, Germany). Primers were designed by PSQ Assay Design (Biotage AB, Uppsala, Sweden). Genomic DNA was modified by sodium bisulfite using the EZ DNA Methylation Kit (Zymo Research, Irvine, CA, USA) according to the manufacturer’s instructions [48]. In hepatocyte nuclear factor 4 A (HNF4A), the CpG sites are located from 159,936,862 to 159,937,373 bp of chromosome 3q42 region (reference sequence was GENBANK No. NC_005102.4, Figure S5). Additionally, these sites include exons of HNF4A isoform 1 (NM_022180.2). The primers used for the analysis via bisulfite amplicon sequencing are represented in Table S1. Amplification was carried out using the following cycling protocol; a cycle of 95 °C for 4 min; 35 cycles of 95 °C for 30 s, 55 °C for 30 s, and 72 °C for 30 s; and finally 72 °C for 7 min. The PCR products were purified using QIAquick PCR columns (Qiagen, Hilden, Germany) and quantified using Picogreen (Invitrogen, Carlsbad, CA, USA).

### 4.6. Gene Expression Analysis by Quantitative Real-Time PCR

From the liver tissue of the male offspring, total RNA was isolated using the Trizol reagent (Invitrogen, Carlsbad, CA, USA) according to manufacturer’s instruction. Next, 200 units of SuperScript ™ III reverse transcriptase (Invitrogen) was used to perform reverse transcription in 20 μL reaction mixtures containing 1 μg of RNA. A quantitative real-time polymerase chain reaction (qRT-PCR) was performed using an ABI PRISM 7000 sequence detection system (Applied Biosystems, Foster City, CA, USA). Each 20 μL volume of reaction mixture contained 1 μL of complementary deoxyribonucleic acid (cDNA), 10 μL SYBER Premix EX Taq (Takara Bio, Shiga, Japan), and 200 nM primer. The PCR proceeded at 95 °C for 10 min, followed by 40 cycles at 95 °C for 15 s; annealing at 62 °C for 1 min; and a dissociation stage of 1 cycle at 95 °C for 15 s, 62 °C for 20 s, and 95 °C for 15 s. The expression levels of each target gene were calculated using the cycle threshold value method (ΔΔCT), which normalized the values against the CT values of beta-actin (β-actin). The primers used for qRT-PCR are represented in Table S2.

### 4.7. Protein Expression Analysis by Western Blot

Total protein was extracted from the liver of 3-month-old male offspring using RIPA buffer (Boseasang, Seongnam, Korea) containing 1× Protease Inhibitor Cocktail (Roche Diagnostics GmbH, Mannheim, Germany), and the supernatants were collected by centrifugation (16,600× *g* for 30 min at 4 °C). The concentration of protein was determined with a BCA protein assay kit (Sigma, St Louis, MO, USA). Samples containing 40 μg protein were separated via SDS-PAGE and transferred onto a nitrocellulose membrane (Whatman, Dassel, Germany). The membrane was blocked in 5% skim milk in Tris-buffered saline (TBS) with 0.01 % Tween-20 for 2 h. The membrane was probed with anti-HNF4A (1:100; Santa Cruz Biotechnology, Santa Cruz, CA, USA), anti-phospho-HNF4A (1:1000; Abcam, Cambridge, UK), anti-sterol regulatory element-binding protein 1 (SREBP1, 1:500; Santa Cruz Biotechnology), anti-phospho-SREBP1 (1:500; Abcam), anti- fatty acid synthesis (FASN, 1:1000; Santa Cruz Biotechnology), anti-microsomal triglyceride transfer protein (MTTP, 1:1000; Santa Cruz Biotechnology), anti-phosphoenolpyruvate carboxykinase (PEPCK, 1:1000; Santa Cruz Biotechnology), anti-glucose-6-phosphatase (G6Pase, 1:1000; Santa Cruz Biotechnology), and anti-β-actin (1:1000; Santa Cruz Biotechnology). A suitable secondary antibody was used for the final step of protein expression. The membrane bands were developed using an enhanced chemiluminescence reagent (Amersham Pharmacia Biotech, Piscatway, NJ, USA). The expression levels were normalized and expressed as the fold change relative to the β-actin expression.

### 4.8. Statistical Analysis

Data were expressed as the means ± standard error of mean (SEM) and were analyzed using SPSS 24.0 software (Chicago, IL, USA). The three groups were compared using one-way analysis of variance (ANOVA) followed by Tukey’s post-hoc test (*p* < 0.05). *p* < 0.05 was considered statistically significant.

## 5. Conclusions

In conclusion, in our study, maternal food restriction or a high-fat diet during prenatal and postnatal periods affected the dysregulation of hepatic metabolism among male offspring at 3 months of age. These results suggest that maternal diet could predispose individuals to type 2 diabetes and non-alcoholic fatty liver disease in later life through changes in taurine levels and HNF4A methylation.

## Figures and Tables

**Table 1:** Characteristics of male offspring at 3 months of age.

	Control (*n* = 9)	FR/AdLib (*n* = 9)	HF/HF (*n* = 9)
Birth weight (g)	7.09 ± 0.06	6.30 ± 0.08 ^†^	6.66 ± 0.07 ^‡,^*
Weight (g)	466.78 ± 5.83	445.50 ± 10.96	602.11 ± 17.65 ^‡,^*
Weight gain (%)	6474.33 ± 82.07	6971.42 ± 173.99 ^†^	8886.71 ± 263.41 ^‡,^*
Liver (g)	14.18 ± 0.28	14.27 ± 0.74	17.17 ± 0.68 ^‡,^*
Liver/Weight (%)	3.06 ± 0.06	3.18 ± 0.07	2.85 ± 0.05 ^‡,^*
Epididymal fat pad (g)	4.90 ± 0.27	5.01 ± 0.56	15.58 ± 1.62 ^‡,^*

Effects of maternal food restriction and high-fat diet during pregnancy on male offspring at 3 months of age. Values are presented as the mean ± SEM. *p*-values were calculated using one-way analysis of variance (ANOVA) by Tukey’s post-hoc test. ^†^ Control vs. FR/AdLib, ^‡^ Control vs. HF/HF, * FR/AdLib vs HF/HF (*p* < 0.05). AdLib, ad libitum; FR, food restriction; HF, high fat.

**Table 2:** Plasma metabolic profiles in male offspring at 3 months of age.

	Control (*n* = 9)	FR/AdLib (*n* = 9)	HF/HF (*n* = 9)
**Plasma metabolic profiles**	-	-	-
Glucose (mg/dl)	142.22 ± 7.20	173.38 ± 11.61 ^†^	174.67 ± 7.17 ^‡^
Insulin (ng/mL)	0.15 ± 0.03	0.14 ± 0.01	0.38 ± 0.06 ^‡,^*
HOMA-IR	1.31 ± 0.21	1.79 ± 0.18 ^†^	4.02 ± 0.58 ^‡,^*
Total cholesterol (mg/dl)	59.38 ± 2.82	63.13 ± 3.68	82.33 ± 5.38 ^‡,^*
HDL -cholesterol (mg/dl)	30.00 ± 5.28	43.75 ± 6.04	46.11 ± 10.08
LDL -cholesterol (mg/dl)	6.00 ± 0.66	6.38 ± 0.53	7.44 ± 1.12
Triglyceride (mg/dl)	58.22 ± 8.24	63.63 ± 13.86	62.44 ± 8.62

Effects of maternal food restriction and a high-fat diet during pregnancy on the plasma metabolic profiles of male offspring at 3 months of age. Values are presented as the mean ± SEM. *p*-values were calculated using one-way analysis of variance (ANOVA) followed by Tukey’s post-hoc test. ^†^ Control vs. FR/AdLib, ^‡^ Controls vs. HF/HF, * FR/AdLib vs. HF/HF (*p* < 0.05). AdLib, ad libitum; FR, food restriction; HDL, High-density lipoprotein; HF, high fat; HOMA-IR, Homeostasis model assessment for insulin resistance, calculated as (fasting plasma glucose [mmol/L] × fasting insulin [µIU/mL])/22.5; LDL, Low-density lipoprotein.

**Table 3:** HNF4A methylation in the liver of male offspring at 3 months of age.

HNF4A-P1_3M					
	Control	FR/AdLib	HF/HF	Control vs. FR/AdLib (*p*-Value)	Control vs. HF/HF (*p*-Value)
CpG1	0.2 ± 0.02	0.31 ± 0.04	0.31 ± 0.04 ^‡^	0.059	**0.025**
CpG2	0.2 ± 0.05	0.31 ± 0.04 ^†^	0.32 ± 0.04 ^‡^	**0.028**	**0.016**
CpG3	0.21 ± 0.01	0.32 ± 0.04 ^†^	0.33 ± 0.04 ^‡^	**0.028**	**0.027**
CpG4	0.21 ± 0.02	0.32 ± 0.04 ^†^	0.33 ± 0.04 ^‡^	**0.002**	**0.019**
CpG5	0.21 ± 0.02	0.33 ± 0.04 ^†^	0.32 ± 0.04 ^‡^	**0.02**	**0.025**
CpG6	0.21 ± 0.02	0.31 ± 0.04 ^†^	0.31 ± 0.04 ^‡^	0.055	**0.033**
CpG7	0.16 ± 0.02	0.25 ± 0.03 ^†^	0.28 ± 0.03 ^‡^	**0.047**	**0.007**
CpG8	0.21 ± 0.02	0.33 ± 0.04 ^†^	0.35 ± 0.04 ^‡^	**0.031**	**0.01**
CpG9	0.16 ± 0.02	0.25 ± 0.03	0.27 ± 0.03 ^‡^	0.058	**0.011**
CpG10	0.2 ± 0.02	0.31 ± 0.04	0.33 ± 0.04 ^‡^	0.088	**0.028**

Effects of maternal diet during pregnancy on HNF4A methylation in the liver of male offspring at 3 months of age. Values are presented as the mean ± SEM. *p*-values were calculated using one-way analysis of variance (ANOVA) followed by Tukey’s post-hoc test. _†_ Control vs. FR/AdLib, _‡_ Control vs. HF/HF (*p* < 0.05). AdLib, ad libitum; FR, food restriction; HF, high fat; HNF4A, hepatocyte nuclear factor 4A. Bold font is used to emphasize *p*-value significant below 0.05.

## Supplementary Materials

Supplementary materials can be found at https://www.mdpi.com/1422-0067/21/23/9060/s1.
