# Supplementary material for: Maternal Malnutrition Affects Hepatic Metabolism through Decreased Hepatic Taurine Levels and Changes in HNF4A Methylation
Source: Int J Mol Sci. 2020 Nov 28;21(23):9060. doi: 10.3390/ijms21239060 (PMC7729756; doi:10.3390/ijms21239060)
Supplement: Supplementary file 1 [file ijms-21-09060-s001.pdf]

Maternal malnutrition affects hepatic metabolism through decreased hepatic taurine levels and changes in HNF4A methylation

Ji Eun Du[1], Young-Ah You[1], Eun Jin Kwon[1], Soo Min Kim[1], Jeongae Lee[2], Ki-Hwan Han[3], Young Ju Kim[1].

1. Department of Obstetrics and Gynecology and Ewha Medical Research Institute, College of Medicine, Ewha Womans University, Seoul 07985, Korea.

2. Molecular Recognition Research Center, Korea Institute of Science and Technology, Seoul 02792, Korea.

3. Department of Anatomy, College of Medicine, Ewha Womans University, Seoul 07985, Korea

Running title: Changes in hepatic metabolism by maternal diet

Corresponding Author

Young Ju Kim, Department of Obstetrics and Gynecology and Ewha Medical Research Institute, Ewha Womans University Medical School, Seoul 07985, Korea. Tel: +82-2-2650-5029, Fax: +82-2-2647-9860, E-mail: kkyj@ewha.ac.kr

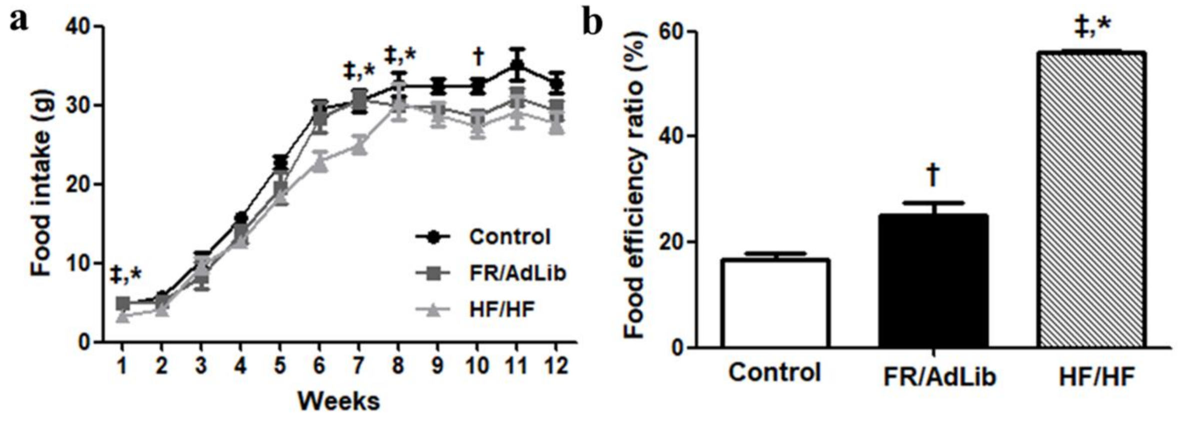

Figure. S1 Food Efficiency in male offspring at 3 months of age. a Daily food intake (g) **b** Food efficiency ratio (FER). Food efficiency ratio calculated as (body weight (g) / food intake (g)) x 100. Values are presented as the mean ± SEM. P values were calculated using one-way analysis of variance (ANOVA) followed by Tukey's post-hoc test. †Control vs FR/AdLib, ‡Controls vs HF/HF, * FR/AdLib vs HF/HF ($p<0.05$). AdLib, ad libitum; FR, food restriction; HF, high fat.

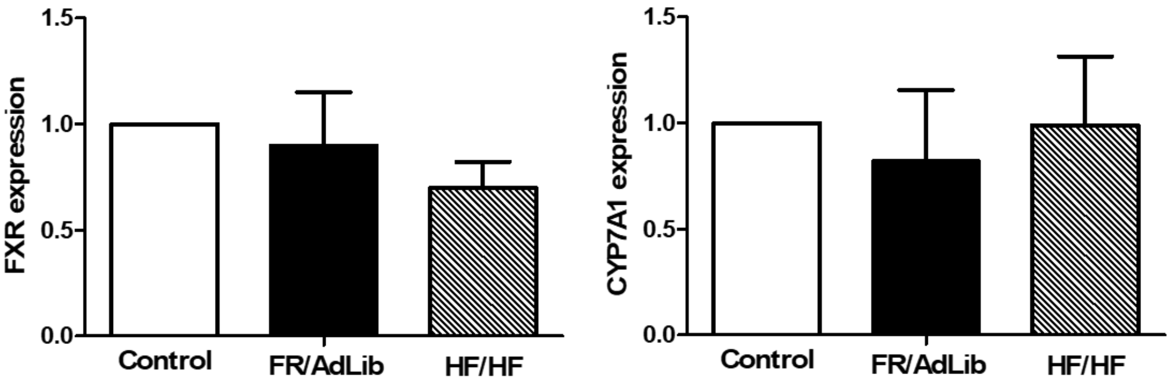

Figure. S2 Relative mRNA expression of FXR and CYP7A1 in the liver of male offspring at 3 months of age. The levels of mRNA encoding FXR and CYP7A1 were determined by real-time PCR in the three groups (n=9 control, n=9 FR/AdLib, n=9 HF/HF). Values are presented as the mean ± SEM. P values were calculated using one-way analysis of variance (ANOVA) followed by Tukey's post-hoc test. AdLib, ad libitum; FR, food restriction; HF, high fat; FXR, farnesoid X receptor; CYP7A1, Choeterol 7 alpha-hydroxylase

a

ASSAY	ANALYSIS	UNIT
NUTRIENTS		
MOISTURE(OVEN)	12.25	%
PROTEIN(PROTEIN ANALYZER)	21.08	%
FAT(ACID HYDROLYSIS)	5.81	%
FIBER(ANKOM)	2.67	%
ASH(FURNACE)	6.38	%
CALCIUM(AAS)	1.14	%
PHOSPHORUS(COLORMETRIC)	0.61	%
HEAVY METALS		
As(ICP)	0.23	ppm
Cd(ICP)	0.02	ppm
Hg(MERCURY ANALYZER)	1.13	ppb
Pb(ICP)	Not detected	ppm
Se(ICP)	0.13	ppm
AFLATOXIN(ELISA)		
B1, B2, G1, G2	Not detected	ppb
CHLORINATED HYDROCARBON		
DDT(GC)	Not detected	ppm
ORGANOPHOSPHATES		
MALATHION(GC)	Not detected	ppm
Microbial Tests		
Salmonella	Not detected	mm
Total Bacteria	450	cfu/g
E. Coli	Not detected	cfu/g
PHYSICAL PROPERTIES		
PELLET SIZE(DxL)	16.70*13.06	mm
PELLET COLOR	Brown	-
HARDNESS	14.3	kg/cm^2

b

Product #	D12451	
	gm%	kcal%
Protein	24	20
Carbohydrate	41	35
Fat	24	45
Total		100
kcal/gm	4.73	
**Ingredient**	**gm**	**kcal**
Casein, 80 Mesh	200	800
L-Cystine	3	12
Corn Starch	72.8	291
Maltodextrin 10	100	400
Sucrose	172.8	691
Cellulose, BW200	50	0
Soybean Oil	25	225
Lard*	177.5	1598
Mineral Mix S10026	10	0
DiCalcium Phosphate	13	0
Calcium Carbonate	5.5	0
Potassium Citrate, 1 H2O	16.5	0
Vitamin Mix V10001	10	40
Choline Bitartrate	2	0
FD&C Yellow Dye #5		
FD&C Red Dye #40	0.05	0
FD&C Blue Dye #1		
**Total**	**858.15**	**4057**

Figure. S3 Composition of Diet. a composition of standard laboratory chow. **b** composition of 45% high-fat diet.

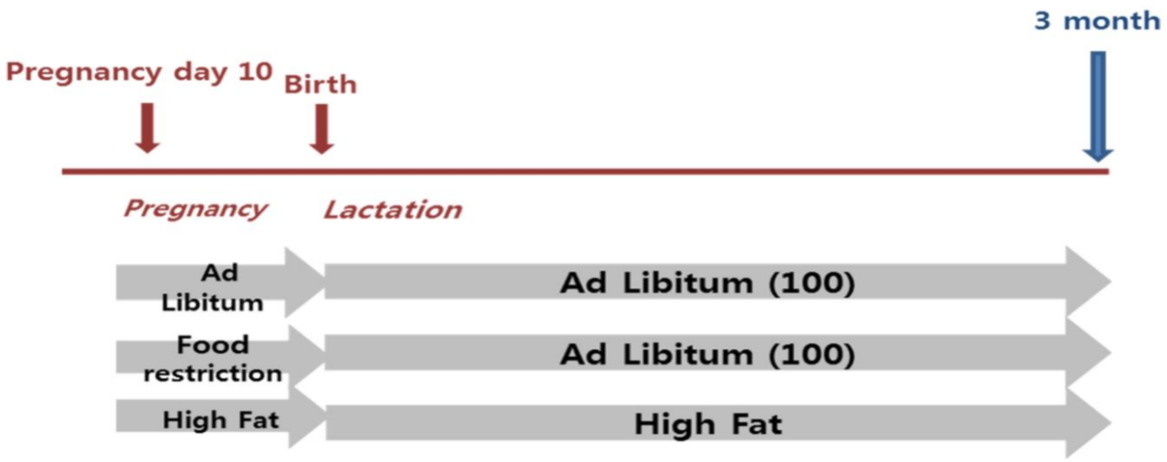

Figure. S4 Experimental design. Eight-week-old Sprague-Dawley (SD) rats were used in this study. Pregnant rats were divided randomly into three groups: 1) AdLib/AdLib for control (fed ad libitum during entire experimental period); 2) FR/AdLib (given a 50% food-restriction diet during pregnancy and ad libitum during lactation); 3) HF-HF (given a 45% high fat diet during pregnancy and lactation).

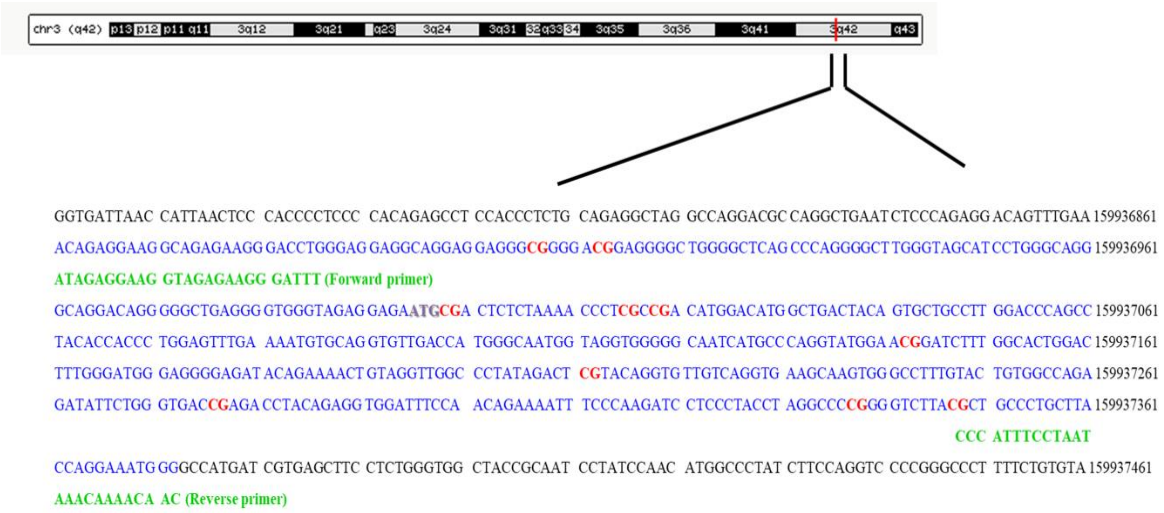

Figure. S5 Schematic representation of the CpG sites in the HNF4α gene. In HNF4A, the CpG sites are located from 159,936,862 to 159,937,373 bp of chromosome 3q42 region. HNF4α; Hepatocyte nuclear factor 4 α

Table S1. Primer sequences of gene for Bisulfite Amplicon Sequencing

Genes	Sequences (5'-3')	Length (base pair)
HNF4α	Forward : ATAGAGGAAGGTAGAGAAGGGATTT<br>Reverse : CCCATTTCCTAATAAACAAAACAAC	512 bp

HNF4α; Hepatocyte nuclear factor 4 α

Table S2. Primer sequences for Quantitative real-time PCR of the mRNA expression

Gene (Accession Number)		Primer sequences
HNF4α (NM_022180.2)	Forward	AAATGTGCAGGTGTTGACCA
	Reverse	CACGCTCCTCCTGAAGAATC
SREBP1(XM_213329.6)	Forward	GGAGCCATGGATTGCACATT
	Reverse	AGGAAGGCTTCCAGAGAGGA
FASN (NM_017332.1)	Forward	TGGCTTCCGTTCAGTCTCTT
	Reverse	CAGTGCCAAGGTCTCTAGCC
MTTP (NM_001107727.1)	Forward	AAGGCCAATATGGACATCCAGGGT
	Reverse	TGGTTATTACCACAGCCACCCGAT
PEPCK (NM_198780.3)	Forward	GTGTCATCCGCAAGCTGAAG
	Reverse	CTTTCGATCCTGGCCACATC
G6Pase (NM_013098.2)	Forward	CCCAGACTAGAGATCCTGACAGAA
	Reverse	GCACAACGCTCTTTTCTTTTACC
β-actin	Forward	AGGGAAATCGTGCGTGAC
	Reverse	CGCTCATTGCCGATAGTG

HNF4α, Hepatocyte nuclear factor 4α; SREBP1, Sterol regulatory element-binding protein 1; FASN, Fatty acid synthesis; MTTP, Microsomal triglyceride transfer protein; PEPCK, Phosphoenolpyruvate carboxykinase; G6Pase, glucose-6-phosphatase; β-actin, Beta-actin.
